# Novel Family of Gynecologic Cancer Antigens Detected by Anti-HIV Antibody

**DOI:** 10.1155/S1064744994000608

**Published:** 1994

**Authors:** Ewa M. Rakowicz-Szulczynska, David G. McIntosh, Peter Morris, McClure Smith

**Affiliations:** 1 University of Nebraska Medical Center Department of Obstetrics and Gynecology 600 South 42nd Street Omaha NE 68198-3255 USA; 2 Eppley Cancer Institute Omaha NE USA

## Abstract

*Objective:* The reactivity of gynecologic cancer proteins with monoclonal antibody (MAb) directed against the human immunodeficiency virus I (HIV-I) was tested.

*Methods:* Cytoplasmic and nuclear proteins, extracted from a broad range of gynecologic cancers obtained during standard surgical procedures, were tested in Western blotting with MAb 5023 developed against the amino acid sequences 308–322 of the envelope protein gp120 of HIV-I.

*Results:* Three cell membrane proteins, M_r_l20,000 (p120), M_r_41,000 (p41), and M_r_24,000 (p24), and one chromatin protein, M_r_24,000 (p24), were detected by MAb 5023 in invasive, poorly differentiated cervical squamous-cell carcinoma; ovarian serous cystadenocarcinoma; poorly and well-differentiated endometrial carcinoma; vulvar squamous-cell carcinoma; and malignant mixed müllerian tumor. The same antigens were identified in cervical carcinoma cell line SiHa. Neither p120 nor p24 was recognized by other MAbs directed against the variable loop of gp120. Antigens p120 and p41 were undetectable in normal ovarian tissue and in biopsy samples of normal vaginal and rectal mucosa. Rectosigmoid cancer as well as colon carcinoma, lung carcinoma, and melanoma cell lines all tested negative.

*Conclusions:* The identified antigens may represent either the products of human genes (proto-onc-ogenes) or, more likely, the products of an unknown virus specifically expressed in female cancer.


Infectious Diseases in Obstetrics and Gynecology 2:171–178 (1994)
© 1994 Wiley-Liss, Inc.

### Novel Family of Gynecologic Cancer Antigens Detected by Anti-HIV Antibody

**Ewa M. Rakowicz-Szulczynska, David G. McIntosh, Peter Morris, and McClure Smith**

*University of Nebraska Medical Center, Department of Obstetrics and Gynecology (E.M.R.-S., D.G.M., P.M., M.S.) and Eppley Cancer Institute (E.M.R.-S., M.S.), Omaha, NE*

**Abstract**

***Objective:*** **The reactivity of gynecologic cancer proteins with monoclonal antibody (MAb) directed against the human immunodeficiency virus I (HIV-I) was tested.**

***Methods:*** **Cytoplasmic and nuclear proteins, extracted from a broad range of gynecologic cancers obtained during standard surgical procedures, were tested in Western blotting with MAb 5023 developed against the amino acid sequences 308–322 of the envelope protein gp120 of HIV-I.**

***Results:*** **Three cell membrane proteins, $M_r$120,000 (p120), $M_r$41,000 (p41), and $M_r$24,000 (p24), and one chromatin protein, $M_r$24,000 (p24), were detected by MAb 5023 in invasive, poorly differentiated cervical squamous-cell carcinoma; ovarian serous cystadenocarcinoma; poorly and well-differentiated endometrial carcinoma; vulvar squamous-cell carcinoma; and malignant mixed müllerian tumor. The same antigens were identified in cervical carcinoma cell line SiHa. Neither p120 nor p24 was recognized by other MAbs directed against the variable loop of gp120. Antigens p120 and p41 were undetectable in normal ovarian tissue and in biopsy samples of normal vaginal and rectal mucosa. Rectosigmoid cancer as well as colon carcinoma, lung carcinoma, and melanoma cell lines all tested negative.**

***Conclusions:*** **The identified antigens may represent either the products of human genes (proto-oncogenes) or, more likely, the products of an unknown virus specifically expressed in female cancer.**
© 1994 Wiley-Liss, Inc.

Key words
Cancer diagnosis, retroviral antigens, proto-oncogenes

Cancer of the female genital tract accounts for almost 80,000 cases of invasive cancer each year in the United States, with the majority of these being 1 of 3 neoplasms: carcinoma of the cervix, endometrial carcinoma, and epithelial carcinoma of the ovary.[1] Ovarian carcinoma and breast carcinoma together account for one-third of all cancers occurring in women and are responsible for approximately one-quarter of cancer-related deaths in females.[1–3] Inherited forms of ovarian and breast cancer may frequently[4–6] be associated with the genetic locus BRCA1[7] or with mutations in other genes like p53[8] or Her2/erbB2[9] or estrogen receptors.[10] Since most gynecologic cancer patients do not exhibit any known genetic abnormalities, the etiology of these cancers remains unclear.

Due to the retroviral origin of several cancers in other vertebrates,[11] a search for cancer virus in humans was performed in several laboratories.[12–14] However, except for cervical cancer which is definitely linked with human papillomavirus infection,[15,16] the viral etiology of female malignancies has never been confirmed.

On the other hand, 50% of human immunodeficiency virus (HIV)-infected women develop cervical cancer, which has a more virulent course in HIV-infected than in noninfected women.[17–22] Immunodeficiency, which follows HIV infection, was

Address correspondence/reprint requests to Dr. Ewa M. Rakowicz-Szulczynska, Department of Obstetrics and Gynecology, Univ. of Nebraska Med. Ctr., 600 South 42nd Street, Omaha, NE 68198-3255.

Received June 7, 1994

Accepted August 17, 1994

Clinical Study presented as an explanation for the unusually fast progression of cervical cancer in these patients.[22]

We report that HIV-cross-reactive antigens are expressed in several gynecologic cancers and may represent novel markers of female malignancies. Since many other cancer-associated antigens are also expressed in much smaller quantities on normal cell counterparts, the identification of novel, cancer-specific targets[23–25] is necessary for effective cancer diagnosis and immunotherapy.

### SUBJECTS AND METHODS

### Gynecologic Cancer

Samples of ovarian, cervical, endometrial, vulvar, and mixed müllerian tumors were obtained during standard surgical procedures. The histologic structure of each cancer was determined by the Department of Pathology of the University of Nebraska Medical Center. Rectosigmoid cancer, a nongynecologic cancer, was obtained from the Department of Pathology and used as a negative control. Normal vaginal and rectal mucosa and normal ovarian tissue were collected during the surgery of gynecologic cancer, and the normal histology of these samples was assessed by the Department of Pathology. A small-bowel nodule was surgically removed from a patient with Sertoli-Leydig cell tumor of the ovary.

### Cell Lines

The SiHa cervical carcinoma cell line was obtained from the American Type Tissue Culture Collection and grown in Eagle's minimal essential medium/Leibovitz's L15 medium (3:4) supplemented with 10% fetal bovine serum.

### Monoclonal Antibodies

Monoclonal antibodies (MAbs) 5023 and 5025 (against HIV-1 gp120 amino acid region 308–322)[25,26] are from the DuPont Company. Iodination of MAbs was routinely performed by the IODOGEN method, as described.[25] The specific activity of MAb was 10–20 cpm/pg.

### Tissue Fractionation

The cancer and normal tissue samples obtained during surgery were extensively washed with phosphate-buffered saline (PBS) and pressed through a cell-separating mesh sieve (Sigma). Cells were centrifuged (600 *g* for 5 min), washed 3 times with PBS, and tested by the light microscope. Ovarian cancer was free of erythrocytes, while cervical, endometrial, and vulvar cancer contained 4–7% of erythrocytes. The cells were homogenized in 0.35 M sucrose/10 mM KCl/1.5 mM $MgCl_2$/10 mM Tris-HCl (pH 7.6)/0.12% Triton X-100/12 mM 2-mercaptoethanol (approximately 1 ml/0.5 $cm^3$ of the cancer tissue, with the exception of cervical cancer, which was 1 ml/0.25 $cm^3$) and centrifuged at 600 *g* for 10 min. The supernatant was defined as the cytoplasmic fraction (crude, membrane-containing). In some experiments, the cytoplasm was centrifuged for another 30 min at 10,000 *g* to remove mitochondria and then for 1 h at 100,000 *g* to obtain the microsomal (plasma-membrane) fraction. The nuclear pellet was first washed with 0.2 M sucrose/3 mM $CaCl_2$/50 mM Tris-HCl (pH 7.6) and then with 0.14 M NaCl/10 mM Tris-HCl (pH 8.3) and centrifuged at 700 *g* for 10 min. The pellet was swollen in a small amount of 1 mM Tris-HCl (pH 7.9) and centrifuged through 1.7 M sucrose, 10 mM Tris-HCl (pH 7), at 160,000 *g* for 80 min. Chromatin was pelleted at the bottom of the tube.

### Electrophoresis of Proteins

Nuclear, chromatin, cytoplasmic, and membrane proteins were analyzed by electrophoresis in 7.5–10% polyacrylamide gel with 0.1% sodium dodecyl sulfate (SDS) in buffer containing 250 mM Tris-HCl (pH 8.3), 195 mM glycine, and 0.1% SDS, according to Laemmli et al.[27] Gels were run at 100 V for 3–4 h. The molecular weights of proteins were estimated using high-range and low-range prestained protein markers from Bio-Rad.

### Western Blotting

Blotting of proteins from the polyacrylamide gel to the polyvinylidene difluoride (PVDF) membrane was performed in the Tris-glycine (25 mM Tris-HCl (pH 8.6), 192 mM glycine buffer, containing 10% methanol. Transfer onto the PVDF membrane was performed at 50 V overnight. Membranes were washed with water, followed by Tris-glycine buffer. Filters were incubated with 1% bovine serum albumin (BSA) for 16 h at 0°C, then incubated with MAb 5023 or 5025 (2 μg/ml), washed with Tris-glycine buffer, and incubated with alkaline phosphatase-conjugated goat anti-mouse IgG for 1 h. After being washed with the

Tris-glycine buffer, the membranes were incubated with 0.1% 1-naphthyl-phosphate and Fast Red, in 100 mM Tris-HCl (pH 9.5), 100 mM NaCl, 5 mM $MgCl_2$.

### RESULTS

### Reactivity of Gynecologic Cancer-Associated Antigens With MAb Against HIV-I gp120

MAb 5023 directed against HIV-I gp120 was tested in Western blotting for reactivity with cytoplasmic, plasma-membrane, nuclear, and chromatin proteins of ovarian, cervical, endometrial, and vulvar cancer and mixed müllerian tumor obtained during surgery. To eliminate a possibility of MAb 5023 cross-reactivity with cells other than malignant, which are present in the cancer isolated from the patient, the cervical cancer cell line SiHa was used in the same set of experiments. Three antigens were detected by MAb 5023 in the cytoplasmic/plasma-membrane fraction of all gynecologic cancer types: high molecular weight proteins of $M_r$120,000 (p120), $M_r$41,000 (p41), and $M_r$24,000 (p24) (Fig. 1A–C). The number of cancer samples that tested positive for particular antigens are shown in Table 1. All samples tested positive for p24 and most were also positive for p120, p41, or both. Another MAb directed against HIV-I gp120, MAb 5025, reacted very weakly with $M_r$41,000 antigen (Fig. 1D), but not with other antigens.

### *Cervical Cancer*

In a model experiment with cervical cancer cell line SiHa, the 3 antigens p120, p41, and p24 were detected by MAb 5023 in the cytoplasm and the antigens p41 and p24 in the chromatin (Fig. 1A, lanes 5, 6).

MAb 5023 reacted with all cervical cancer samples obtained from 9 different patients; however, the expression of particular antigens varied. All cancer samples were histologically analyzed and described as invasive, poorly differentiated squamous-cell carcinomas. Cervical cancer CI (Fig. 1, lane 1) and CII (not shown) expressed a well-seen cytoplasmic antigen p41 and a much weaker p24, but did not express p120. Cervical cancer sample CIII expressed only 1 MAb 5023-reactive protein, p24 (Fig. 1, lane 2). In cancer CIV, MAb 5023 detected 2 antigens, p120 and p24, but did not detect p41 (Fig. 1A, lane 3). In cancer CV (Fig. 1A, lane 4) and CVI, CVII, CVIII, and CIX (not shown), all 3 antigens (p120, p41, and p24) were detected. Due to the small size of the cervical cancer, separation of the nucleus from the cytoplasm was not always possible, and total extracts of cervical cancer CIV and CV (lanes 3, 4) were analyzed. However, since p120 is expressed in neither the chromatin of the SiHa cell line (Fig. 1A, lane 6) nor the chromatin of other gynecologic cancers (Fig. 1B,C), strictly cytoplasmic localization of p120 may be established. In addition to the 3 major antigens migrating in the gel with the same mobility as the antigens expressed by the cell line SiHa (Fig. 1A, lane 5), a few protein bands of lower molecular weights (<24,000) were detected in some cervical cancer samples (Fig. 1A, lanes 1, 4). Whether these low molecular weight proteins represent degradation products was not established.

### *Ovarian Cancer*

Antigen p120 was detected by MAb 5023 in the cytoplasm of all tested ovarian cancer samples (Fig. 1B, OII-lane 3, OIII-lane 4, OIV-lane 6, OV not shown, OVII-Fig. 3, lane 1), except for cancer obtained from patient OI (Fig. 1B, lane 1) and OVI (not shown). Cancer OI was histologically characterized as poorly differentiated papillary serous adenocarcinoma involving the left fallopian tube and ovary. Ovarian cancer OVI was histologically described as mucinous adenocarcinoma. The other cancers (OII–V) were described as typical serous cystadenocarcinomas of the ovary with the exception of OVII, which was the endometrioid carcinoma of the ovary.

Antigen p120 was undetectable in the nuclear fractions (Fig. 1B, lanes 2, 5, 7). The $M_r$120,000 band visible in Figure 1B, lane 2, represents impurities attached from the cytoplasm to the nucleus during cell fractionation. After purification of the chromatin, the $M_r$120,000 band was not visible (not shown). Antigen p41 was detected as a weak band in all ovarian cancer samples, but it was detected at a relatively high level in the nucleus of ovarian cancer OIII.

Antigen p24 was visible in the cytoplasm and in the nucleus of all ovarian cancer samples. Similarly, as in cervical cancer (Fig. 1A), antigen p24 exhibited a clear association with both cytoplasmic and nuclear fraction (Fig. 1B, lanes 1–7). In the cytoplasm, an antigen of $M_r$ ~20,000 frequently

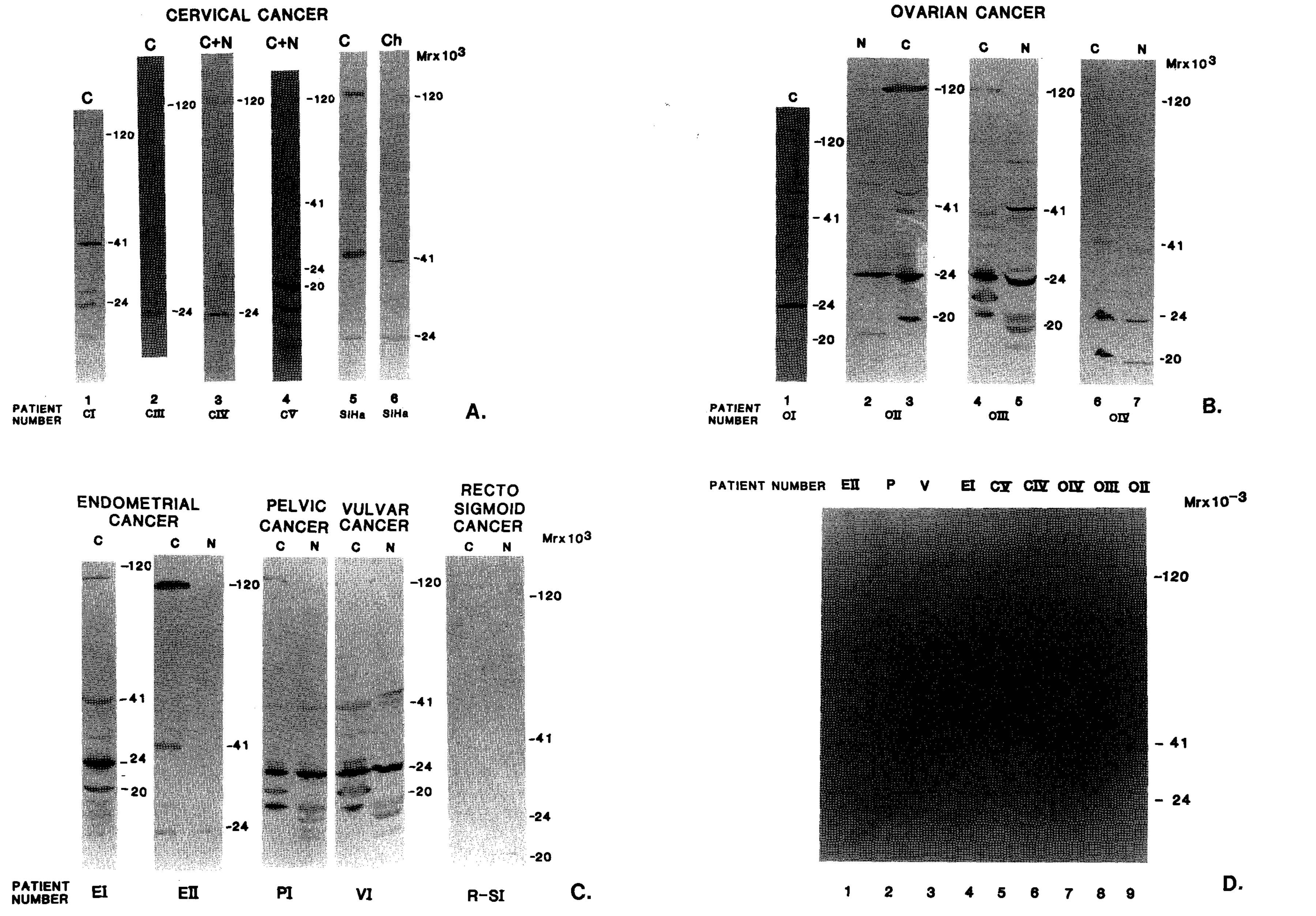

CERVICAL CANCER

C

C

C+N

C+N

C

Ch

Mrx10³

-120

-41

-24

-20

PATIENT NUMBER

1 CI

2 CIII

3 CIV

4 CV

5 SiHa

6 SiHa

A.

OVARIAN CANCER

N

C

C

N

C

N

1 OI

2 3 OII

4 5 OIII

6 7 OIV

B.

ENDOMETRIAL CANCER

PELVIC CANCER

VULVAR CANCER

RECTO SIGMOID CANCER

EI

EII

PI

VI

R-SI

C.

PATIENT NUMBER EII P V EI CV CIV OIV OIII OII

Mrx10⁻³

1 2 3 4 5 6 7 8 9

D.

Fig. 1.

**TABLE 1. Expression of HIV-1 cross-reactive antigens in gynecologic cancer**

| Cancer type | Antigens detected by MAb anti-HIV-1 gp120[a] | No. of patients tested/no. tested positive |
|---|---|---|
| Cervical | p120 | 9/7 |
| | p41 | 9/7 |
| | p24 | 9/9 |
| Ovarian | p120 | 7/5 |
| | p41 | 7/7 |
| | p24 | 7/7 |
| Endometrial | p120 | 4/4 |
| | p41 | 4/4 |
| | p24 | 4/4 |
| Mixed müllerian tumor | p120 | 3/3 |
| | p41 | 3/3 |
| | p24 | 3/3 |
| Vulvar | p120 | 1/1 |
| | p41 | 1/1 |
| | p24 | 1/1 |

[a]The antigens were identified by Western blot analysis of the cytoplasmic fraction with MAb anti-HIV-1 gp120. Each experiment was repeated 4–7 times with 100% reproducibility of the results.

followed p24 (Fig. 1B, lanes 3, 4, 6). In the nuclear fraction, the protein of $M_r$20,000 was undetectable, but instead a few $M_r$ < 20,000 bands were visible. Due to the low molecular weights of these bands, we cannot exclude the possibility that these bands represent the products of protein degradation.

### *Endometrial Cancer*

In endometrial cancer EII, which according to the histologic analysis represented poorly differentiated adenocarcinoma, all 3 cytoplasmic antigens (p120, p41, p24) and p24 in the chromatin (Fig. 1C, lanes EII-C, N) strongly reacted with MAb 5023. The reactivity of MAb 5023 with p120 was stronger than with other antigens in the same tissue and also stronger than in any other tested cancer (Fig. 1A–C). In contrast to p120, p24 was much weaker than in cervical cancer (Fig. 1A), ovarian cancer (Fig. 1B), mixed müllerian tumor, or vulvar cancer (Fig. 1C). The second endometrial cancer, EI (Fig. 1C, EI), which histologically represented well-differentiated adenocarcinoma, also expressed all 3 antigens. However, the reaction of p120 with MAb 5023 was weaker, and the reaction of p24 was stronger than in EII. Antigen p20 followed p24. The nucleus contained only p24 (not shown). Two other patients with advanced endometrial cancer (not shown) showed the same pattern as patient EII.

Fig. 1. Western blotting of MAb 5023 **(A–C)** and of the control MAb 5025 **(D)** directed against HIV-1 gp120, with cytoplasmic (C), nuclear (N), total (C+N), or chromatin (Ch) fractions isolated from particular cancer samples. The sample marked as pelvic cancer (C) was analyzed prior to the histologic examination and represents mixed müllerian tumor. Proteins were separated in 7.5–12% polyacrylamide gel. Different migration is due to the different time of electrophoresis and a different gel density. Prestained protein markers (high and low molecular weight) were used in each experiment to determine the molecular weights of the antigens. Rectosigmoid cancer in C is shown as a negative control, i.e., a nongynecologic cancer.

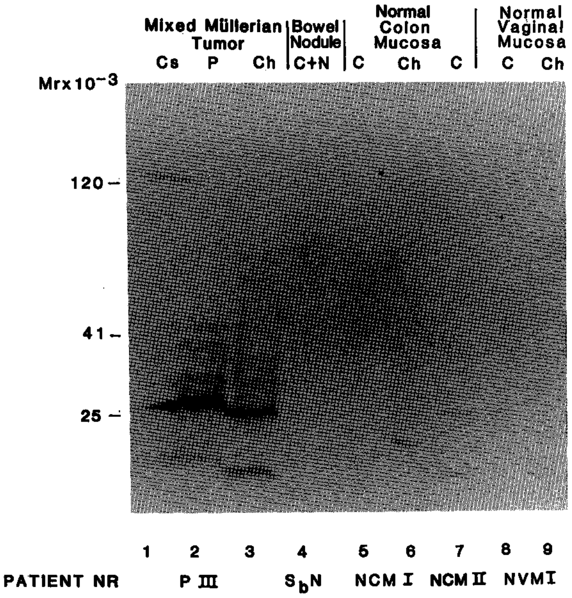

Fig. 2. Western blotting of MAb 5023 with cytosolic **(lane 1)**, microsomal **(lane 2)**, and chromatin **(lane 3)** fractions of mixed müllerian tumor (PIII), nonmalignant nodule from the small bowel **(lane 4)**, normal colon mucosa **(lanes 5–7)**, and vaginal mucosa **(lanes 8, 9)**. C, cytoplasm; Cs, cytosol; P, microsomal fraction; Ch, chromatin; C + N, extract from nonfractionated cells.

### Mixed Müllerian Tumor

Three mixed müllerian tumors were tested. All 3 antigens, p120, p41, and p24 (Fig. 1C, PI), reacted in Western blotting with MAb 5023. Other samples (PII not shown) and PIII (Fig. 2) showed exactly the same pattern as PI.

### *Vulvar Cancer*

Vulvar cancer (invasive squamous-cell carcinoma) showed 3 protein bands (p120, p41, and p24) in the cytoplasm as well as p41 and p24 (Fig. 1C, lanes VI-C and VI-N, respectively) in the nucleus.

### Cytosolic Localization of Antigen p120

The experiments shown in Figure 1 were done with the crude, membrane-containing cytoplasmic fraction. In order to determine whether the antigens recognized by the anti-HIV-I MAb 5023 exhibit plasma-membrane or cytosolic localization, the cytoplasm isolated from the mixed müllerian tumor of patient PIII was split into pure cytosol, microsomal, and chromatin fractions (see Methods section). Electrophoretic analysis followed by Western blotting showed that p120 was localized selectively in the cytosolic fraction (Fig. 2, lane 1), while p24 was mainly detected in the microsomal fraction and in the chromatin (Fig. 2, lanes 2, 3).

### Specificity of MAb 5023 Binding to Gynecologic Cancer Antigens

Cancers other than gynecologic cancer did not react with MAb 5023. Rectosigmoid cancer, fractionated into cytoplasmic and nuclear fractions, did not react with MAb 5023 at the $M_r$120,000 and $M_r$41,000 region. Very weak bands were detected in the cytoplasm and in the chromatin at the $M_r$24,000 region (Fig. 1C, R-SI). MAb 5023 also did not react with a broad range of colorectal carcinoma, lung carcinoma, and melanoma cell lines (not shown).[24] A nodule isolated from the small bowel of the patient with a Sertoli-Leydig cell tumor of the ovary, which was histologically characterized as fibrous tissue, also tested negative in Western blotting with MAb 5023 (Fig. 2, lane 4). Normal colon mucosa obtained from 2 different patients (Fig. 2, lanes 5–7) and vaginal mucosa (Fig. 2, lanes 8, 9) also did not react with MAb 5023 except for a weak reaction with the $M_r$24,000 protein.

The normal ovarian tissue biopsy from patient OVII (Fig. 3, lane 2) did not show reactivity with MAb 5023 except for reactivity with the $M_r$24,000 protein, although cancer isolated from the same patient (Fig. 3, lane 1) exhibited a strong reactivity of p120 and p41 with MAb 5023.

In general, the HIV-cross-reactive antigens p120 and p41 were found to be specifically expressed in gynecologic cancer but were not expressed in normal tissues or in other tumors.

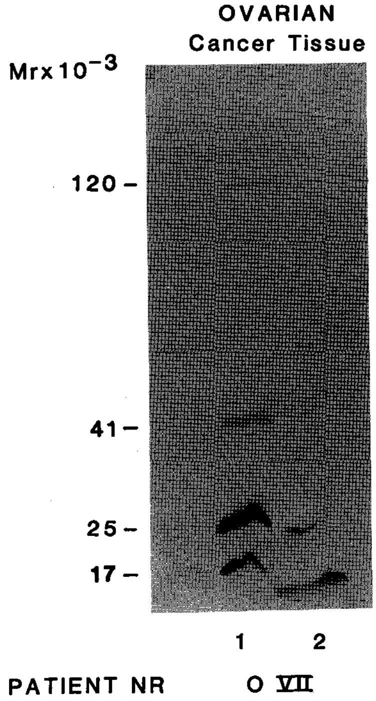

**Fig. 3.** Western blotting of MAb 5023 with the cytoplasmic fraction from ovarian cancer (**lane 1**) and normal ovarian tissue (**lane 2**) from the same patient OVII.

### DISCUSSION

MAb 5023 developed against amino acid residues 308–322 of the variable V3 loop of the HIV-1 envelope protein gp120 was found to cross-react with antigens p120, p41, and p24 in a broad range of gynecologic cancers, including squamous-cell carcinoma of the cervix, ovarian serous cystadenocarcinoma, endometrial cancer, malignant mixed müllerian tumor, and vulvar cancer. Antigen p120 was selectively localized in the cytoplasm, while p41 and p24 were localized in the cytoplasm and the nucleus.

In addition to the reactivity with gynecologic cancer, MAb 5023 exhibits strong binding to breast carcinoma cell antigens p160/p80, p45, and p24.[24] Since the molecular weight of p160 in breast cancer cells corresponds to the molecular weight of the precursor for HIV envelope proteins, and the molecular weight of p120 in gynecologic cancer corresponds to the molecular weight of the HIV envelope antigen gp120, an accidental homology of short epitopes is highly unlikely. Since MAb directed against HIV-I gp120 is effectively internalized by breast cancer[24] and cervical cancer cells, which leads to stimulation of cell proliferation (Rakowicz-Szulczynska, unpublished data), a growth-factor receptor-like character of HIV-cross-reactive cancer antigens may be anticipated. The fact that HIV-cross-reactive antigens are expressed in various gynecologic cancers of different histologic structures would support the growth-factor receptor-like character of these proteins. On the other hand, the HIV-1-cross-reactive antigens were undetectable in colorectal carcinoma, lung carcinoma, and melanoma cell lines, which makes a simple growth-factor receptor-like character of these proteins highly unlikely. We suggest that an unknown retrovirus with some homology to HIV-1 might be expressed in gynecologic cancer. Very recently, we have obtained results with the polymerase chain reaction (PCR) confirming that long regions with 100% homology to HIV-I are present in gynecologic cancer DNA (Rakowicz-Szulczynska, unpublished data).

HIV-cross-reactive antigens p120 and p41 seem to be selectively expressed by cancer cells. Normal ovarian tissue, vaginal mucosa, and colon mucosa did not express p120 or p41. It is also noteworthy that rectosigmoid cancer (Fig. 1C), as well as several melanoma, colorectal carcinoma, and lung carcinoma cell lines tested previously,[24] do not express HIV-cross-reactive antigens p120 and p41. Thus, these antigens are specifically associated with breast[24] and gynecologic cancer. In contrast to p120 and p41, p24 was found in some other cancer cell lines,[24] as well as in normal ovarian tissue and in noninfected lymphocytes.[25] Protein p24 cannot be considered a cancer-associated antigen.

MAb 5023 was developed against a short polypeptide (RIQRGPGRAFVTIGK), corresponding to the amino acids 308–322 of HIV-1 gp120, but this MAb binds to the much shorter amino acid region GRAF.[26] It is likely that this core epitope is expressed in gynecologic cancer and breast cancer antigens. Another MAb, MAb 5025, which was developed against the same synthetic peptide but recognizes the sequences RAF, did not bind to gp120 and p24, but reacted weakly with p41 (Fig. 1D). It is noteworthy that a fraction of human antibodies isolated from HIV-infected people also recognized in Western blotting breast cancer[24] and gynecologic cancer (unpublished data) antigens. The results suggest that HIV-infected patients produce antibodies that cross-react with cancer. The biological function and diagnostic value, as well as the genetic origin, of the HIV-cross-reactive cancer antigens remain to be further investigated.

### ACKNOWLEDGMENTS

This work was sponsored by the Leland J. and Dorothy H. Olson Center for Women's Health Foundation. The authors thank Monica Strauss for help in manuscript preparation.

### REFERENCES

1. Silverberg E, Boring CC, Squires TS: Cancer statistics. CA 40:9–15, 1990.
2. Aure JC, Hoeg K, Kolstad P: Clinical and histologic studies of ovarian carcinoma: Long-term follow-up of 990 cases. Obstet Gynecol 37:1–7, 1971.
3. Bjorkholm E, Pettersson E, Einhorn N, Krebs I, Nilsson B, Tjernberg B: Long-term follow-up and prognostic factors in ovarian carcinoma: The Radiumhemmet series 1958 to 1973. Acta Radiol 21:413–417, 1982.
4. King MC, Rowell S, Love SM: Inherited breast and ovarian cancer. JAMA 269:1975–1980, 1993.
5. Biesecker BB, Boehnke M, Calzone K, Markel DS, Garber JE, Collins FS, Weber BL: Genetic counseling for families with inherited susceptibility to breast and ovarian cancer. JAMA 269:1970–1974, 1993.
6. Newman B, Austin MA, Lee M, King MC: Inheritance of human breast cancer: Evidence for autosomal dominant transmission in high-risk families. Proc Natl Acad Sci USA 85:3044–3048, 1988.
7. Narod SA, Feunteun J, Lynch HT, Watson P, Conway T, Lynch J, and Lenoir GM: Familial breast-ovarian cancer locus on chromosome 17q12-23. Lancet 338:82–83, 1991.
8. Coles C, Condie A, Chetty U, Steel CM, Evans HJ, Prosser J: p53 mutations in breast cancer. Cancer Res 52:5291–5298, 1992.
9. Slamon DJ, Godolphin W, Jones LA, Holt JA, Wong SG, Keith DE, Levin WJ, Stuart SG, Udove J, Ullrich A, Press MF: Studies of the HER-2/neu proto-oncogene in human breast and ovarian cancer. Science 244:707–712, 1989.
10. Zuppan PJ, Hall JM, Lee MK, Ponglikitmongkol M, King MC: Possible linkage of the estrogen receptor genes to breast cancer in a family with late-onset disease. Am J Hum Genet 48:1065–1068, 1991.
11. Pimentel E (ed): Hormones, Growth Factors, and Oncogenes, Boca Raton: CRC Press, pp 1–19, 1987.
12. Gardner MB, Rasheed S, Shimizu S, Rongey RW, Henderson BE, McAllister RW, Klement V, Charman P, Gilden RV, Heberling RL, Huebner RJ: Search for RNA tumor viruses in humans, Cold Spring Harbor Conference. Cell Prolif 4:1235–1251, 1977.

13. Ilyin KV, Bykovsky AF, Zhdanov VM: An oncoRNAvirus isolated from human cancer cell line. Cancer 32:89–96, 1973.
14. Feller WF, Chopra HC: A small virus-like particle observed in human breast cancer by means of electron microscopy. J Natl Cancer Inst 40:1359–1373, 1968.
15. Iwasaka T, Yokoyama M, Oh-Uchida M, Matsuo N, Hara K, Fukuyama K, Hachisuga T, Fukuda K, Sugimori H: Detection of human papilloma virus genome and analysis of expression of c-myc and Ha-ras oncogenes in invasive cervical carcinomas. Gynecol Oncol 46:298–303, 1992.
16. Hwang JY, Lin BY, Tang FM, Winston CY: Tamoxifen stimulates human papilloma virus type 16 gene expression and cell proliferation in a cervical cancer cell line. Cancer Res 57:6848–6852, 1992.
17. Schafer A, Schwartlande RB, Friedman W: Epidemiology and clinical aspects of the HIV-infected female. Arch Gynecol Obstet 245:173–178, 1989.
18. Rogo KO, Kavoo-Linge: Human immunodeficiency virus seroprevalence among cervical cancer patients. Gynecol Oncol 37:87–92, 1990.
19. Carpenter CC, Mayer KH, Stein MD, Leibman BD, Fisher A, Fiore TC: Human immunodeficiency virus infection in North American women: Experience with 200 cases and a review of the literature. Medicine 70: 307–325, 1991.
20. Williams AB: The epidemiology, clinical manifestations and health-maintenance needs of women infected with HIV. Nurse Pract 17:27, 31–34, 37–38, 1992.
21. Schafer A, Friedmann W, Mielke M, Schwartlander B, Koch MA: The increased frequency of cervical dysplasia-neoplasia in women infected with the human immunodeficiency virus is related to the degree of immunosuppression. Am J Obstet Gynecol 164:593–599, 1991.
22. Matorras R, Ariceta JM, Rementeria A, Corral J, Gutierrez-de-Teran G, Diez J, Montoya F, Rodriguez-Escudero FJ: Human immunodeficiency virus-induced immunosuppression: A risk factor for human papilloma virus infection. Am J Obstet Gynecol 164:42–44, 1991.
23. Rakowicz-Szulczynska EM: Nuclear antigens as the new targets in cancer therapy. In Rakowicz-Szulczynska EM (ed): Nuclear Localization of Growth Factors and of Monoclonal Antibodies. Boca Raton: CRC Press, pp 129–178, 1993.
24. Rakowicz-Szulczynska EM, Kaczmarski W, Steimer KS, Durda PJ: Internalized antibodies as a potential tool against retroviral disease. In Rakowicz-Szulczynska EM (ed): Nuclear Localization of Growth Factors and of Monoclonal Antibodies. Boca Raton: CRC Press, pp 129–197, 1993.
25. Rakowicz-Szulczynska EM, Kaczmarski W, Raso V, Steimer KS, Durda PJ: Internalization of anti-gp120 monoclonal antibody and human antibodies by HIV-1-infected lymphocytes. Antibody Immunoconjugates Radiopharmaceut 6:209–219, 1993.
26. Langedijk JPM, Back NKT, Durda PJ, Goudsmit J, Melsen RH: Neutralizing activity of anti-peptide antibodies against the principal neutralization domain of human immunodeficiency virus type 1. J Gen Virol 72: 2519–2526, 1991.
27. Laemmli UK: Cleavage of structural proteins during the assembly of the head of bacteriophage T4. Nature 227: 680–685, 1971.

## References

[PDF_00527] Aure J. C., Hoeg K., Kolstad P. (1971). Clinical and histologic studies of ovarian carcinoma. Long-term follow-up of 990 cases.. Obstet Gynecol.

[PDF_00536] Biesecker B. B., Boehnke M., Calzone K., Markel D. S., Garber J. E., Collins F. S., Weber B. L. (1993). Genetic counseling for families with inherited susceptibility to breast and ovarian cancer.. JAMA.

[PDF_00530] Björkholm E., Pettersson F., Einhorn N., Krebs I., Nilsson B., Tjernberg B. (1982). Long-term follow-up and prognostic factors in ovarian carcinoma. The radiumhemmet series 1958 to 1973.. Acta Radiol Oncol.

[PDF_00591] Carpenter C. C., Mayer K. H., Stein M. D., Leibman B. D., Fisher A., Fiore T. C. (1991). Human immunodeficiency virus infection in North American women: experience with 200 cases and a review of the literature.. Medicine (Baltimore).

[PDF_00548] Coles C., Condie A., Chetty U., Steel C. M., Evans H. J., Prosser J. (1992). p53 mutations in breast cancer.. Cancer Res.

[PDF_00572] Feller W. F., Chopra H. C. (1968). A small virus-like particle observed in human breast cancer by means of electron microscopy.. J Natl Cancer Inst.

[PDF_00581] Hwang J. Y., Lin B. Y., Tang F. M., Yu W. C. (1992). Tamoxifen stimulates human papillomavirus type 16 gene expression and cell proliferation in a cervical cancer cell line.. Cancer Res.

[PDF_00569] Ilyin K. V., Bykovsky A. F., Zhdanov V. M. (1973). An oncornavirus isolated from human cancer cell line.. Cancer.

[PDF_00534] King M. C., Rowell S., Love S. M. (1993). Inherited breast and ovarian cancer. What are the risks? What are the choices?. JAMA.

[PDF_00630] Laemmli U. K. (1970). Cleavage of structural proteins during the assembly of the head of bacteriophage T4.. Nature.

[PDF_00625] Langedijk J. P., Back N. K., Durda P. J., Goudsmit J., Meloen R. H. (1991). Neutralizing activity of anti-peptide antibodies against the principal neutralization domain of human immunodeficiency virus type 1.. J Gen Virol.

[PDF_00604] Matorras R., Ariceta J. M., Rementeria A., Corral J., Gutierrez de Terán G., Diez J., Montoya F., Rodriguez-Escudero F. J. (1991). Human immunodeficiency virus-induced immunosuppression: a risk factor for human papillomavirus infection.. Am J Obstet Gynecol.

[PDF_00544] Narod S. A., Feunteun J., Lynch H. T., Watson P., Conway T., Lynch J., Lenoir G. M. (1991). Familial breast-ovarian cancer locus on chromosome 17q12-q23.. Lancet.

[PDF_00540] Newman B., Austin M. A., Lee M., King M. C. (1988). Inheritance of human breast cancer: evidence for autosomal dominant transmission in high-risk families.. Proc Natl Acad Sci U S A.

[PDF_00588] Rogo K. O., Kavoo-Linge (1990). Human immunodeficiency virus seroprevalence among cervical cancer patients.. Gynecol Oncol.

[PDF_00599] Schäfer A., Friedmann W., Mielke M., Schwartländer B., Koch M. A. (1991). The increased frequency of cervical dysplasia-neoplasia in women infected with the human immunodeficiency virus is related to the degree of immunosuppression.. Am J Obstet Gynecol.

[PDF_00585] Schäfer A., Schwartländer B., Friedmann W. (1989). Epidemiologie und Klinik der HIV-infizierten Frau.. Arch Gynecol Obstet.

[PDF_00525] Silverberg E., Boring C. C., Squires T. S. (1990). Cancer statistics, 1990.. CA Cancer J Clin.

[PDF_00551] Slamon D. J., Godolphin W., Jones L. A., Holt J. A., Wong S. G., Keith D. E., Levin W. J., Stuart S. G., Udove J., Ullrich A. (1989). Studies of the HER-2/neu proto-oncogene in human breast and ovarian cancer.. Science.

[PDF_00596] Williams A. B. (1992). The epidemiology, clinical manifestations and health-maintenance needs of women infected with HIV.. Nurse Pract.

[PDF_00556] Zuppan P., Hall J. M., Lee M. K., Ponglikitmongkol M., King M. C. (1991). Possible linkage of the estrogen receptor gene to breast cancer in a family with late-onset disease.. Am J Hum Genet.

